# Microstructure and Mechanical Properties of a Novel Al-Mg-Sc-Ti Alloy Fabricated by Laser Powder Bed Fusion

**DOI:** 10.3390/ma17030686

**Published:** 2024-01-31

**Authors:** Zhiheng Shu, Yunzhong Liu

**Affiliations:** National Engineering Research Center of Near-Net-Shape Forming for Metallic Materials, South China University of Technology, Guangzhou 510640, China; zhs2765590059@163.com

**Keywords:** laser powder bed fusion, Al-Mg alloy, Sc/Ti addition, microstructures, mechanical properties

## Abstract

(TiH_2_ + ScH_3_)/Al-Mg composite powders with different Ti contents were produced by ball milling. These composite powders were fabricated to cube and cuboid shape samples via a laser powder bed fusion process with optimal processing parameters. The TiH_2_ and ScH_3_ particles underwent dehydrogenation during the laser powder bed fusion process, and these composite powders ultimately formed Al-Mg-Sc-Ti alloys. The relative density, printability, microstructure, hardness and tensile properties of these alloy samples were investigated. The results show that these Al-Mg-Sc-Ti alloys have lower hot-crack sensitivity, having fine equiaxed grains. An Al_18_Mg_3_(Ti,Sc)_2_ intermetallic phase and in situ L1_2_-Al_3_(Sc,Ti) precipitations formed during the laser powder bed fusion process, which is beneficial for nucleation and dispersion strengthening. The ultimate tensile strength of the Al-Mg-0.7Sc-1.0Ti alloy was 313.6 MPa with an elongation of 6.6%. During the hot isostatic pressing treatment, most of the Mg element precipitated from the matrix and changed the Al_3_(Sc,Ti) into a Al_18_Mg_3_(Ti,Sc)_2_ precipitate completely. The Al-Mg-Sc-Ti alloys were nearly fully dense after the hot isostatic pressing treatment and exhibited better mechanical properties. The ultimate tensile strength of the Al-Mg-0.7Sc-1.0Ti was 475 MPa with an elongation of 8.5%.

## 1. Introduction

Laser powder bed fusion (LPBF) is a dominant technology of additive manufacturing technology that can fabricate parts by spreading and melting powders layer by layer using a focused laser beam [1,2]. Parts with complex structures can be fabricated via LPBF technology without a mold, and the metal powders used by this technology can be reused [3,4]. Unlike other traditional processing methods, the parts fabricated by the LPBF process exhibit a unique microstructure owing to the rapid melting and cooling rates (10^3^~10^6^ K/s) [5,6]. Al-Mg alloys, such as the 5000 series, have the advantages of low density, corrosion resistance and high specific strength [7]. LPBFed Al-Mg alloys have been used in the airplane industry and have a wide range of application prospects in the aerospace field [8]. However, most Al alloys, including 5000 series Al alloys, exhibit low printing performance [1]. These Al alloys have high thermal conductivity and a large freezing range, which makes it easy for them to generate and grow columnar grains during the printing process [9,10]. These columnar grains are normally coarse in shape, which will cause anisotropy of the microstructure and form hot cracks during the printing process [11]. Many studies have delivered solutions to the hot crack problem. Optimizing the process parameters can solve the crack problem for some kinds of Al alloys [12,13], and one of the most common methods is adding grain refiners to the matrix. These grain refiners have the ability of converting the coarse columnar grains to fine exquiaxed grains. These exquiaxed grains are more stable in solidification processing, so they have the benefit of inhibiting the formation of hot cracks. Some researchers use ceramic particles, such as TiB_2_ [14,15] and TiC [16,17], as nucleating agents for aluminum. These TiB_2_/TiC-modified Al alloys exhibit better printability, according to their conclusions. In addition, adding materials that can form intermetallic compounds with Al to the matrix is also an effective method [10,18].

Parts of the grain refiners can react with the matrix in situ to form L1_2_-Al_3_X(X for Sc, Zr, Ti) precipitations [19,20,21]. These Al_3_X precipitations are super-effective nucleating agents for grain refinement during the printing process due to a low lattice misfit with aluminum. Moreover, researchers found that Al alloys co-modified by these X elements exhibit better mechanical properties. Schmidtkes [22] developed a new Sc-/Zr-modified Al alloy for additive manufacturing technology, and the development, production and testing of parts built up by a laser powder bed process has been exhibited in their work. In other studies [23,24], this kind of Al-Mg-Sc-Zr alloy was used in the LPBF process and achieved excellent mechanical properties thanks to the in situ L1_2_-Al_3_(Sc,Zr).

This study aimed to design a new aluminum alloy using the element Ti instead of Zr. The mechanism of replacing Zr with Ti in an Al-Mg-Sc alloy is explained as follows: The element Ti can make the aluminum matrix form a large constitutional supercooling at the solidification front and is beneficial for refining grains and improving mechanical properties [25]. The lattice misfit of L1_2_-Al_3_Ti relative to aluminum is negative, but the lattice misfits of L1_2_-Al_3_Sc and L1_2_-Al_3_Zr relative to aluminum are positive [26]. So the lattice misfit of L1_2_-(Sc,Ti) to aluminum may be lower than L1_2_-(Sc,Zr). The lattice misfit between Al_3_(Sc_0.75_,Ti_0.25_) and aluminum is lower than that between Al_3_(Sc_0.75_,Zr_0.25_) and aluminum, according to the calculation [27]. The room temperature mechanical properties of the Al-Mg-Sc alloy may be improved by adding the element Ti. Developing a new Al-Mg alloy is beneficial for further expanding the application fields of LPBFed Al-Mg alloys.

In this work, TiH_2_ and ScH_3_ particles instead of pure metal powders were chosen to co-modify Al-Mg alloy powders due to their anti-oxidation ability and low costs. This method has higher flexibility compared to using the pre-alloy and can more freely regulate the element ratio in the alloys. During the LPBF process, TiH_2_ and ScH_3_ particles are dehydrogenated and react with the matrix in situ to form Al_3_(Sc,Ti). Parts of the hydrogen are stored by the samples and become gas pores. The hot isostatic pressing (HIP) treatment is conducted to act as a post-process and make these samples fully dense.

## 2. Materials and Methods

### 2.1. Composite Powder Preparation and LPBF Processing

**Figure 1:**
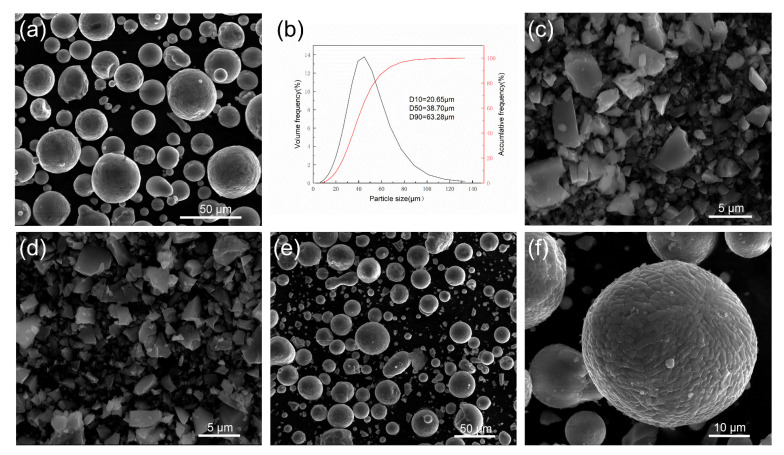
(**a**) Al-Mg alloy powders, (**b**) size distribution of Al-Mg alloy powders, (**c**) ScH_3_ powders, (**d**) TiH_2_ powders, (**e**) (TiH_2_+ScH_3_)/Al-Mg composite powders and (**f**) a magnified section of (**e**).

Al-Mg raw powders were produced by centrifugal atomization after melting in a vacuum induction furnace, and they were basically spherical (Figure 1a). The median particle size of the powders was 38.70 μm (Figure 1b). The elemental composition of the raw Al-Mg powders, excluding Al, was 5% Mg and 0.5% Mn. TiH_2_ (Figure 1c) and ScH_3_ (Figure 1d) particles were prepared using a high-energy ball milling method, and the size distribution of these irregular particles was about 1~10 μm. The corresponding compositions of powders used in this work are shown in Table 1. All the composite powders were prepared via low-energy ball milling under an Argon atmosphere. The ball milling parameters were as follows: the ball-to-material ratio was 5:1, the ball mill speed was 140 rpm and the mixing time was 4 h. Figure 1e,f presents the SEM photographs of the BST2 composite powders. It can be seen that the spherical Al-Mg powders barely changed in shape as a result of the milling process. In addition, composite powders were dried at 100 °C for 8 h in a vacuum dryer before the LPBF process.

**Figure 2:**
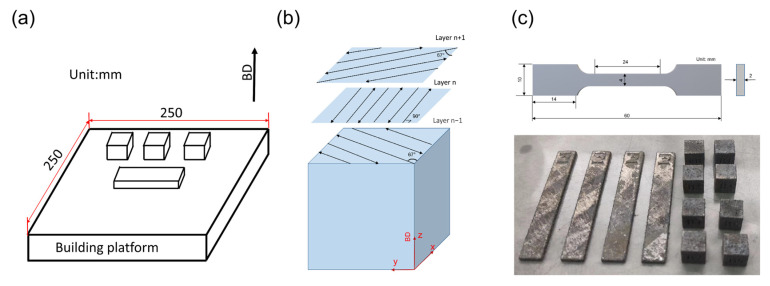
(**a**) Schematic diagram of the LPBF process, (**b**) schematic diagram of the laser scanning strategy, (**c**) process scheme of strip sample in (**a**) and physical drawing of the sample.

The composite powders were loaded in an EOS M280 AM system (EOS GmbH, Krailling, Germany). The preheated temperature of this system was set to 200 °C, and the maximum building zone of this system is 250 × 250 mm^2^ (Figure 2a). The cavity was filled with argon gas during the whole printing process. The laser applied rotated by 67° between adjacent layers following a striped pattern (Figure 2b). The optimized LPBF processing parameters are listed in Table 2. Two kinds of samples were fabricated. The size of cube samples was 10 mm × 10 mm × 10 mm, and the size of strip samples was 65 mm × 10 mm × 2.1 mm. The building direction was perpendicular to the XOZ plane.

### 2.2. Microstructural and Mechanical Characterization

The Keller reagent (95 mL H_2_O + 2.5 mL HNO_3_ + 1.5 mL HCl + 1 mL HF) was used to corrode the cube samples to obtain grain boundary morphology, and a kind of NaOH reagent (0.5 mol/L) was used for the second-phase distribution observation. Pores and cracks on the surface were observed using an optical metallographic microscope (OM). A scanning electron microscope (SEM, Nova Nano430, TMO, Waltham, MA, USA) equipped with energy dispersive X-ray spectroscopy (EDS) was used to detect the morphology of the raw and composite powders, the microstructures of the LPBFed samples and the fracture morphology of the samples after tensile experiments. The voltage of the SEM was set to 15 KV. X-ray diffraction (XRD, D8ADVANCE, Bruker, Germany) using Cu Kα radiation was conducted to scan the XOY surface of the cube samples for phase identification. XRD was performed at a scanning rate of 6°/min with a step size of 0.013°. Electron backscatter diffraction (EBSD, NOVA Nano SEM 430, TMO, MA, USA) was conducted for grain texture and grain size and characterization. The scanning rate of the EBSD was 0.008 μm. A transmission electron microscope (TEM, JEM 2100F, JEOL, Akishima, Japan) was applied for phase identification and microstructural observation. These TEM samples were all prepared using ion thinning. Additionally, a UV–Visible–NIR Lambda 950 PerkinElmer spectrometer based on a diffusion reflectance spectroscopy (DRS) method was employed for measuring the laser reflectivity of raw and composite powders.

The relative density was measured via an Archimedes drainage method. For each alloy, two cube samples and one strip sample were tested to ensure precision. The hardness of these samples was measured on the XOY surface using DHV-1000Zmachine The pressure applied was 0.98 N for 10 s, and 5 points were tested for each sample. Tensile experiments were carried out by an AG-IC 50 KN machine with a strain rate of 0.6 mm/min. Before the tensile tests, the strip samples were machined to bone shapes (Figure 2c), and three samples were tested for each composition.

### 2.3. Hot Isostatic Pressing

Hot isostatic pressing (HIP) was conducted post-process to make the as-fabricated cube and strip samples fully dense. The HIP treatment was conducted on a QUINTUS QIH 15L facility. The whole process was under an Ar atmosphere, and the processing parameters were as follows: the cavity was heated to 450 °C, the pressure applied was 120 MPa and the duration of the process was 2 h.

## 3. Results and Discussion

### 3.1. Cracks and Relative Density

**Figure 3:**
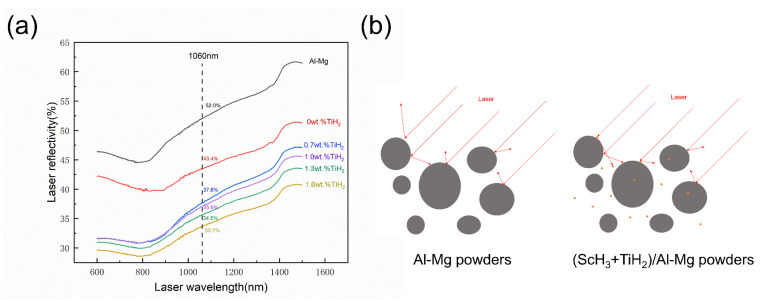
(**a**) The laser reflectivity of the composite powders. (**b**) Schematic diagram of the laser acting on the powders.

Figure 3a shows the laser reflectivity of the raw and composite powders. The raw Al-Mg powders have a laser reflectivity of 52% when the laser wavelength is 1060 nm, which means that only 48% of the input energy was absorbed by the B powders during the printing process. The laser reflectivity of the composite powders decreases with an increase in the particle addition. The BST4 samples have a minimal laser reflectivity of 33.7%. Figure 3b exhibits the schematic diagram of the laser acting on these composite powders.

**Figure 4:**
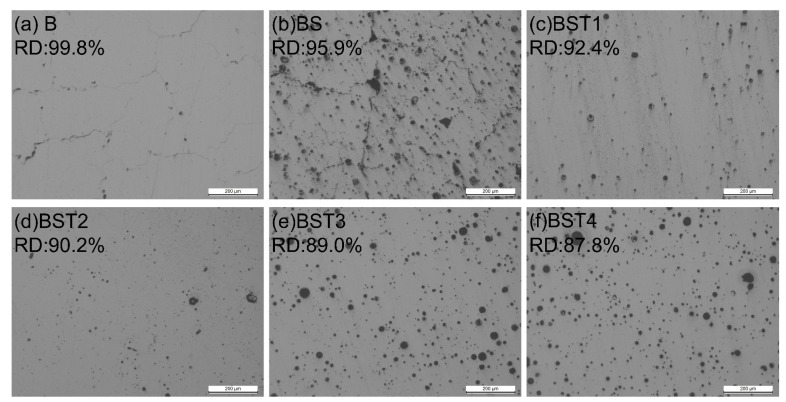
OM images of XOY planes and relative density (RD): (**a**) B sample, (**b**) BS sample, (**c**) BST1 sample, (**d**) BST2 sample, (**e**) BST3 sample, (**f**) BST4 sample.

The micrograph in Figure 4 shows OM images of the as-fabricated samples on the XOZ plane. Cracks exist only in the B and BS samples. The cracks in the B sample are continuous, and cracks induce the formation of keyholes in the BS sample. The pores defect, common in LPBFed alloys [28,29,30], occurs in all these samples. Most of the spherical pores are caused by particle dehydrogenation, while others are related to melting conditions with the metastable state of powders during the printing process. Relative density is a criterion for judging the as-fabricated samples [31]. The relative density of the as-fabricated samples shows that the B samples are nearly fully dense. Obviously, the relative density of these as-fabricated samples decreases with an increase in particle addition. The relative densities of the BST group (including BST1, BST2, BST3 and BST4) samples are lower than 95%, and the BST4 sample has the lowest relative density of 88%. The size and number of these spherical pores increase with the increase in particle addition. The direct effect of pores is a reduction in relative density, which is consistent with the relative density of these samples.

### 3.2. Microstructure and Phase Analysis

**Figure 5:**
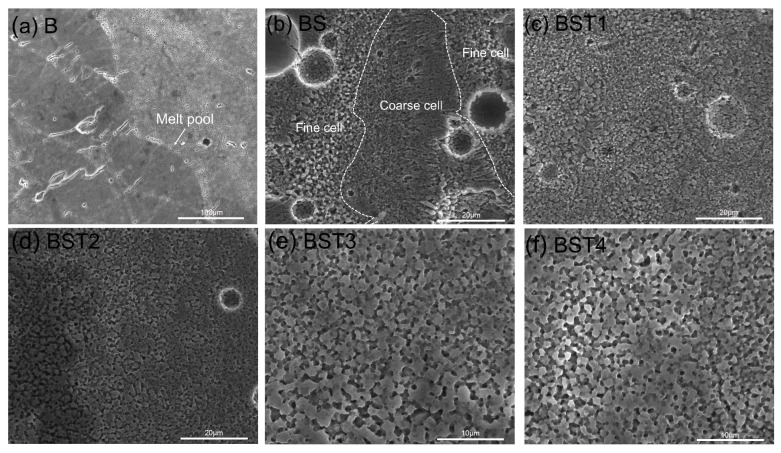
SEM images showing the XOZ plane of samples after etching by the Keller reagent: (**a**) B sample, (**b**) BS sample, (**c**) BST1 sample, (**d**) BST2 sample, (**e**) BST3 sample, (**f**) BST4 sample.

Figure 5 depicts the grain microstructures of these as-fabricated samples on the XOZ plane. As shown in Figure 5a, these molten pools exhibit corrugation, and cracks on the XOZ surface are generated nearly perpendicular to the molten pool. Both coarse columnar grains and fine equiaxed grains exist in the BS samples, and the distribution of these cells is affected by the melt pool boundary (white lines in Figure 5b). Additionally, the growth direction of the columnar grains inside the molten pool is approximately parallel to the building direction. Conversely, the BST group samples have a fine-grain structure without columnar grains. In addition, the pores affected the size of the grains. The results demonstrate that the addition of TiH_2_ and ScH_3_ can convert columnar grains to equiaxed grains in the printing process. Furthermore, the distribution of grains is more uniform and the grain size becomes smaller with the increase in Ti content.

**Figure 6:**
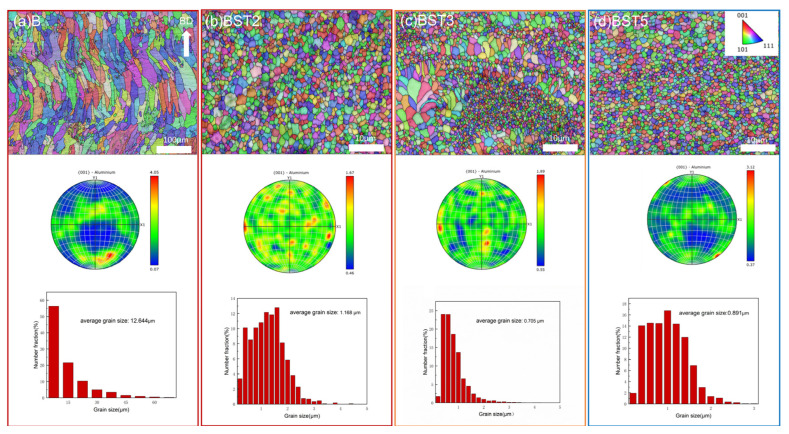
IPF maps, pole figures and grain size distribution images along the building direction of as -fabricated samples: (**a**) B, (**b**) BST1, (**c**) BST2, (**d**) BST3.

Figure 6 shows IPF maps, pole figures and grain size distribution images of the as-fabricated B, BST1, BST2 and BST4 samples across the building direction. Compared with the B sample, the average grain size of these BST group samples is much lower. The BST samples also exhibit lower peak values in the pole figures. The B sample exhibits a certain level of texture, with the maximum texture index as high as 4.05 due to the epitaxial growth of columnar grains, and it can be asserted that the fiber texture is significantly weakened by adding Sc and Ti to the matrix. Additionally, the average grain size of the BST2 sample is about 39.6% lower than that of the BST1 sample. The average grain sizes of the BST2 and BST4 samples are similar, and the difference between them can be explained by selection error. The size of grains is also affected by holes and melt pools.

**Figure 7:**
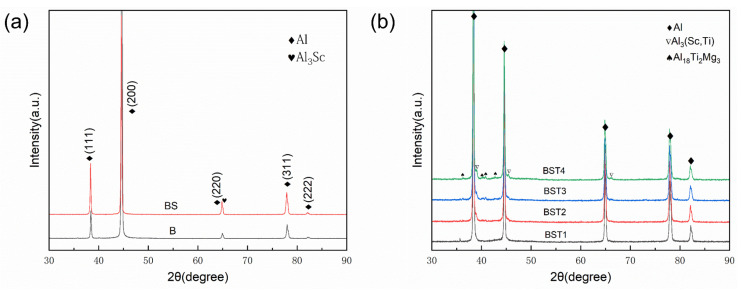
XRD patterns of the as-fabricated samples: (**a**) B and BS samples; (**b**) BST group samples.

Figure 7a shows the XRD patterns of the as-fabricated B and BS samples. The diffraction of Al peaks is clearly visible in these as-fabricated samples. However, the intensity of Al peaks for these samples differs from the standard α-Al phase, which may be caused by the epitaxial growth of columnar grains in these samples. Additionally, the diffraction of the Al_3_Sc peak exists in the BS sample. The XRD patterns of the as-fabricated BST group samples are illustrated in Figure 7b. It can be seen that the diffraction of Al peaks of these samples is consistent with the standard α-Al phase due to the elimination of columnar grains. In addition, peaks of Al_3_(Sc,Ti) and Al_18_Ti_2_Mg_3_ are visible, and the BST4 samples exhibit the highest peak intensity of these phases. The Al_18_Ti_2_Mg_3_ is a common phase in the Al-Mg-Ti alloys, which has been exhibited in other studies [32,33]. Phases containing Mn cannot be found in all samples due to their contents.

**Figure 8:**
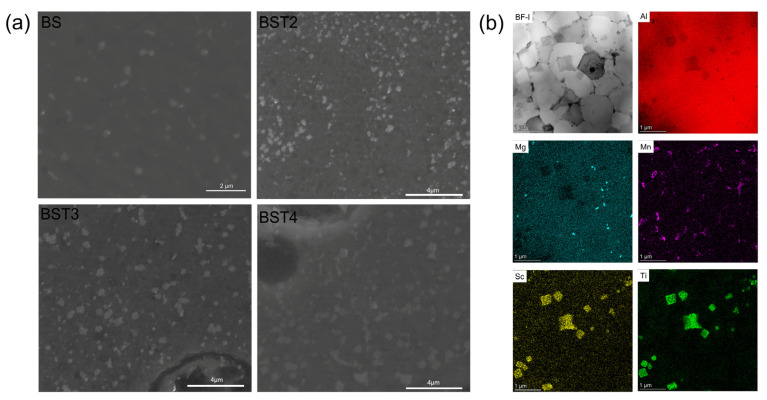
(**a**) SEM images showing the XOZ plane of samples after etching by NaOH reagent; (**b**) TEM results of the as-fabricated BST2 sample and EDS elemental mappings.

A combination of other assays is needed to confirm the results of the phase identification via XRD. Figure 8a shows the shape of precipitates in the as-fabricated samples. The precipitates in the BS sample are assumed to be Al_3_Sc, according to the XRD results, and the precipitates in the BST group samples should be Al_3_(Sc,Ti). Most of these precipitates are irregularly shaped, and only a small number of these precipitates are nearly square. The bright image and corresponding EDS mappings of the BST2 sample are shown in Figure 8b. The results show that only a small amount of Mg and Mn have precipitated from the Al matrix. Mg is basically evenly distributed, and Mn is enriched around the grain boundaries. Obviously, Sc and Ti accumulate in the same precipitates, and these particles are mainly distributed within the grain boundaries.

**Figure 9:**
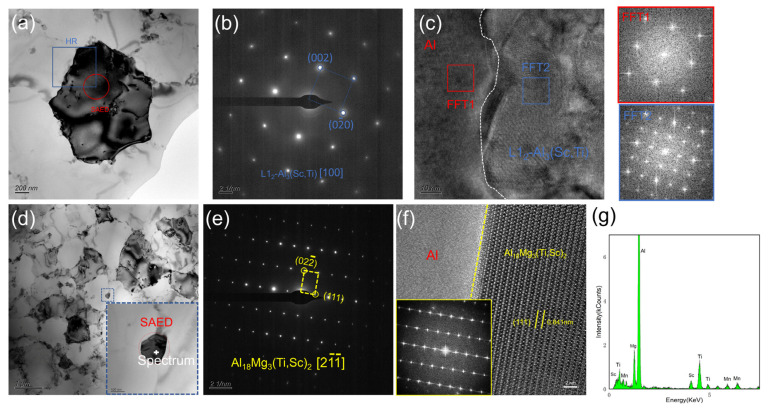
TEM results of the as-fabricated BST2 sample: (**a**) BF image of Al_3_(Sc,Ti) particle, (**b**) SAED pattern in (**a**), (**c**) HRTEM image and corresponding FFTs, (**d**) BF image of Al_18_Mg_3_(Ti,Sc)_2_ particle, (**e**) SAED pattern in (**d**), (**f**) HAADF image and corresponding FFT, (**g**) EDS spectrum in (**d**).

The TEM results of the as-fabricated BST2 sample are given in Figure 9. Figure 9a,d show precipitates with two different morphologies. Selected-area electron diffraction pattern (SAED) and high-resolution (HR) TEM images were conducted for both precipitates. The diffraction pattern (Figure 9b) of the precipitate in Figure 9a was recorded along the [100] zone axis and shows the 002 and 0$\bar{2}$0 type superlattice reflections from the L1_2_ phase. Herein, the precipitate in Figure 8a can be identified as L1_2_-Al_3_Sc or L1_2_-Al_3_Ti. Combined with the previous EDS results (Figure 8b), the precipitate should be identified as L1_2_-Al_3_(Sc,Ti), which is still coherent with the matrix, and the HRTEM results shown in Figure 9c verify that an orientation relationship exists between this precipitate and the matrix. Figure 9e shows the SAED result of a precipitate in Figure 9d. The diffraction pattern was recorded along the [2$\bar{1}\bar{1}$] zone axis and shows the 02$\bar{2}$ and 111 type lattice reflections, which is identified as the Al_18_Ti_2_Mg_3_ phase, but the EDS spectrum of this precipitate exhibits a certain amount of the Sc element. Thus, this precipitate should be identified as Al_18_Mg_3_(Sc,Ti)_2_. The HRTEM result and corresponding FFT result are given in Figure 9f.

### 3.3. Mechanical Properties

**Figure 10:**
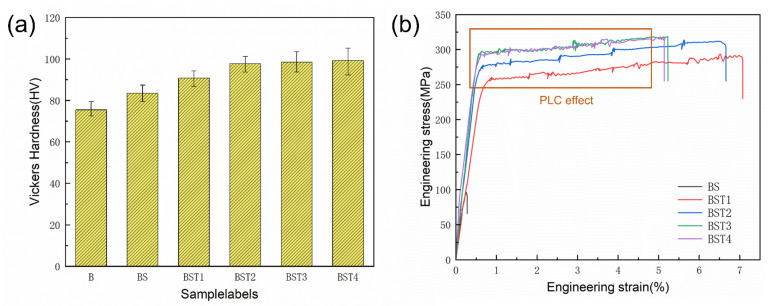
Mechanical properties of the as-fabricated samples: (**a**)Vickers Hardness; (**b**) stress–strain curves of as-fabricated BS and BST group samples.

**Figure 11:**
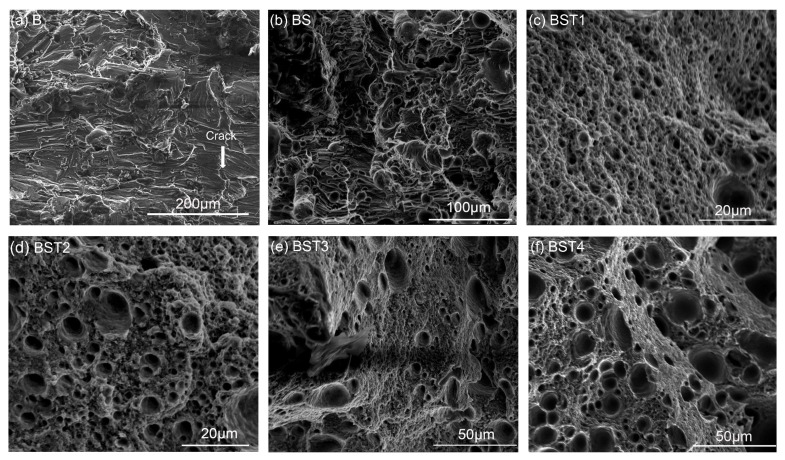
Fracture morphologies of the as-fabricated samples: (**a**) B sample, (**b**) BS sample, (**c**) BST1 sample, (**d**) BST2 sample, (**e**) BST3 sample, (**f**) BST4 sample.

The data in Figure 10a show the microhardness on the XOZ plane of these as-fabricated samples. The average microhardness of the as-fabricated B sample was 75.43 HV and that of the as-fabricated BST4 sample reached 99.25 HV. It is obvious that the addition of these particles could improve the Vickers hardness of samples. The data in Figure 10b show the results of tensile tests. It should be mentioned that the B sample could not finish the tensile tests due to its crack defect. The BS sample broke prematurely, which was also related to the crack defect. Moreover, it is obvious that the tensile properties of the as-fabricated Al-Mg samples were improved by the addition of ScH_3_ and TiH_2_. The tensile strength and elongation of the BST1 sample were 275 ± 8 MPa and 7.2 ± 0.3%, respectively, while the tensile strength and elongation of the BST4 sample were 315 ± 10 MPa and 5.2 ± 0.2%, respectively. Among all the alloys, the BST4 samples had the highest UTS of 315 MPa and the BST1 samples had the highest El of 7.2%. For the BST group samples, plasticity decreased with an increase in the TiH_2_ addition, and the mechanical properties of the BST3 and BST4 samples were very similar. Additionally, an unstable plastic flow existed in all the stress–strain curves of the BST group samples (box area in Figure 10b), which is attributed to the Portevin–Le Chatelier (PLC) effect. This phenomenon is typical for Al-Mg alloys, as pointed out by Mogucheva [34] and Spierings [35]. This effect is caused by the interaction between these diffusing solute Ti/Sc atoms, Al_3_(Sc,Ti)/Al_18_Mg_3_(Ti,Sc)_2_ particles and mobile dislocations during tensile tests. The fracture morphologies of these samples are illustrated in Figure 11. Columnar arms and cracks existed in the B sample (Figure 11a), and a brittle fracture zone appeared. The fractures of the BS sample and the B sample exhibit similar characteristics. As shown in Figure 11c–f, the brittle fracture feature disappears on the fracture surface of the BST group samples. Conversely, fine uniform dimples and precipitated particles at the bottom of the dimples are present on the fracture surface of the BST group samples, indicating a typical ductile fracture mechanism. Another obvious characteristic is that massive spherical pores appear in all the samples except the B sample. These pores caused stress concentration during the tensile test, reducing the plasticity of these samples. The size and number of pores increase with the TiH_2_ content, which could explain the great change in plasticity of the BST group samples.

In summary, the addition of ScH_3_ and TiH_2_ particles to the matrix powders can effectively improve their laser absorption rate, thus reducing the energy required. Additionally, the hot cracks problem of Al-Mg alloys can be resolved via this method. During the LPBF process, Sc and Ti elements in the particles precipitate and react with the Al matrix in situ to generate Al_3_Sc, Al_3_Ti and Al_18_Ti_2_Mg_3_. Unlike the casting process [36], Al_3_Sc and Al_3_Ti, which formed during LPBF, have an L1_2_-type structure. Due to the similar properties of Sc and Ti atoms, either can be replaced by the other, so that the final precipitate phases are Al_3_(Sc,Ti) and Al_18_Mg_3_(Sc,Ti)_2_. This atomic substitution phenomenon is also found in other Al-Mg-Sc-Zr alloys fabricated by LPBF [20,35], and the L1_2_-type structure is still maintained by the Al_3_(Sc,Ti) precipitates. Precipitates with L1_2_-type structures can provide a favorable condition for α-Al nucleation since they have a similar structure to aluminum [17]. Due to the characteristics of rapid melting and solidification during the LPBF process, there is a saturation value in the amount of Ti addition. According to the EBSD results, when the addition of TiH_2_ reaches 1.0%, the grain refinement effect has essentially reached the upper limit in this work. The BST2 sample had a similar hardness and UTS to the BST3 and BST4 samples. According to the Hall–Petch equation [37].

(1) $${\;\Delta \sigma }_{H-P}=\;K\;\left(d^{-1/2}-{d_{0}}^{-1/2}\right)$$

where Δσ_H−P_ is the yield strength, K is a constant that represents the relative strengthening contribution from grain boundaries—which is set to 0.17 MPa·m^1/2^ for Al alloy [31]—and d and d_0_ are the average grain size of the BST2 sample and B sample, respectively. The calculated strength enhancement by the Hall–Petch equation is 154.66 MPa. Moreover, these in situ L1_2_-Al_3_(Sc,Ti) precipitates can deliver dispersion strengthening. However, all the alloy samples exhibit poor plasticity due to the gas pores.

### 3.4. Effect of HIP Treatment

**Figure 12:**
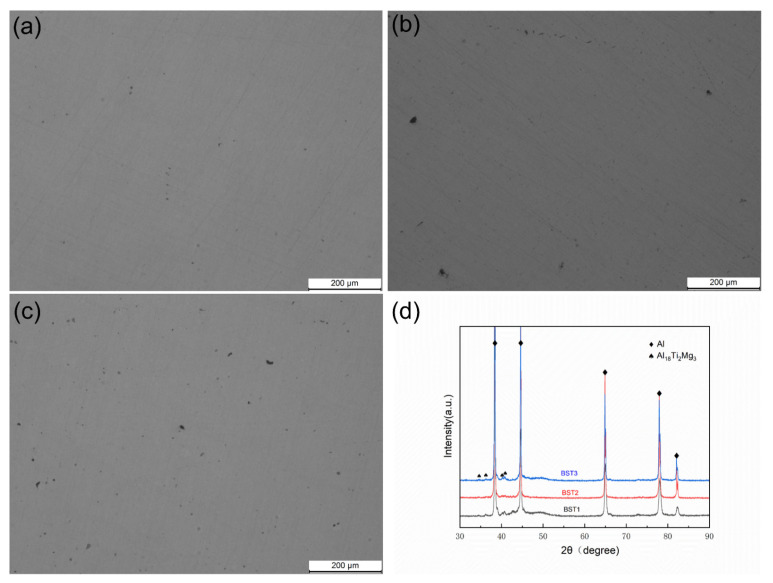
OM images of samples after HIP: (**a**) BST1, (**b**) BST2, (**c**) BST3 and (**d**) XRD patterns of these samples.

**Figure 13:**
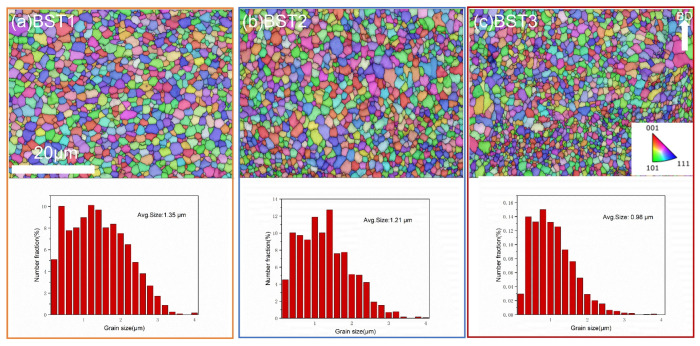
EBSD IPF images of the HIPed samples: (**a**) BST1, (**b**) BST2, (**c**) BST3.

Figure 12 shows OM images on the XOY plane of the samples after the HIP treatment and the XRD pattern of these samples. After HIP, most of the gas pores are eliminated, and the relative density of all these samples is above 99%. The diffraction of the Al peaks barely changes, and the diffraction of the Al_18_Ti_2_Mg_3_ peaks shows a stronger intensity. But the peaks of the Al_3_X phases disappear. The EBSD results of the BST1, BST2 and BST3 samples are given in Figure 13. Compared with the as-fabricated samples, the grain distributions of these HIPed samples is relatively uniform, as the influence of the molten pools and pores was eliminated. The average grain size of the HIPed BST2 sample is 1.21 μm, which is about 71.6% higher than that of the as-fabricated sample. This means that the samples underwent grain growth during HIP treatment, and the content of the Ti element affected the grain growth process, as shown by the reduction in average grain size with the increase in Ti content.

**Figure 14:**
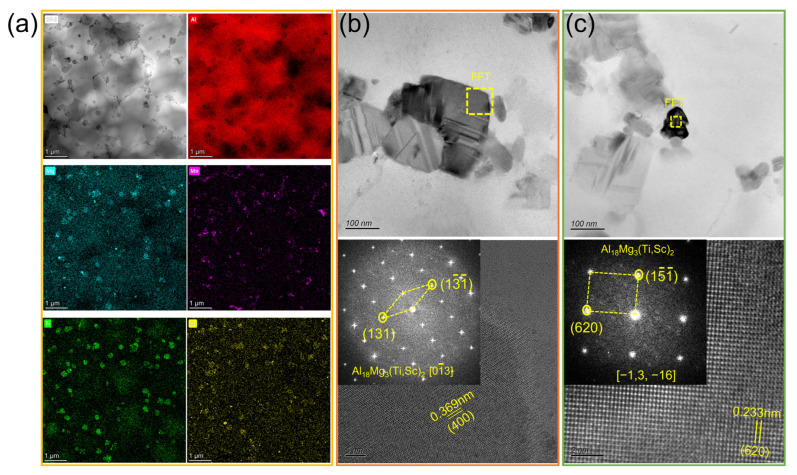
TEM results of the BST2 sample after HIP: (**a**) BF image with corresponding EDS elemental maps; (**b**,**c**) BF images and HRTEM with FFT of Al_18_Mg_3_(Ti,Sc)_2_.

Figure 14 shows the TEM results of the HIPed BST2 sample. Compared with the TEM results of the as-fabricated BST2 sample, it can be deduced that massive Mn and Mg elements precipitate from the matrix during the HIP treatment. Mn still enriches around the grain boundaries. Moreover, the phenomenon that Sc and Ti accumulate in the same zones still exists. Differing from the as-fabricated sample, Mg also accumulates in these zones. There are two distinct precipitates in the matrix, and their morphologies are shown in Figure 14b,c. These precipitates were identified by the HRTEM and the corresponding FFT. According to these results, it can be concluded that these two different precipitates are both Al_18_Mg_3_(Ti,Sc)_2_. Additionally, no orientation relationship exists between these Al_18_Mg_3_(Ti,Sc)_2_ precipitates and the matrix.

**Figure 15:**
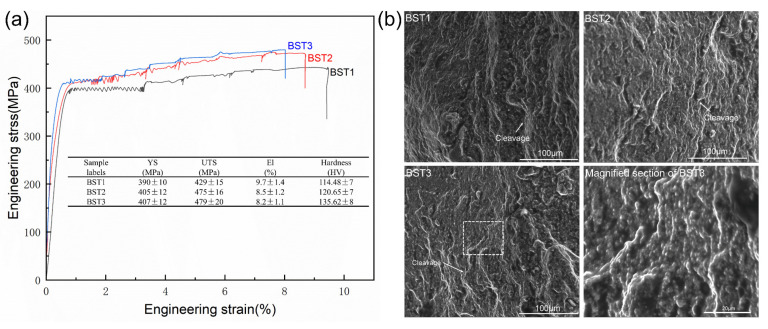
(**a**) Stress–strain curves of HIPed samples, with the inset table showing average values of UTS, YS, El and Hardness; (**b**) fracture morphologies of the samples in (**a**).

Figure 15a shows the SSC curves of these HIPed samples, and the fracture morphologies of these samples are shown in Figure 15b. After HIP treatment, the hardness, plasticity and strength of these alloys have all been improved, and the PLC effect still exists in all these HIPed samples. The mechanical properties and Ti content remained positively correlated. The HIPed BST2 sample has a UTS of 475 MPa, while the El is elevated to 8.5%. Although the plasticity of these materials improved, their fracture mechanisms were all biased toward brittle fractures, and there are river-like features in all these samples (Figure 15b).

Above all, HIP could make the material fully dense and deliver a heat treatment effect similar to solid solution [38]. These as-fabricated Al alloy samples undergo grain growth during the HIP treatment. The element Ti can limit the growth process of grains; samples with higher Ti addition show much lower average grain sizes. As a result of HIP, the average grain size increases and the grain distribution becomes more uniform. The tensile properties, hardness and plasticity of these samples all improved after HIP. The enhancement of these properties is related to phase transitions in these samples. According to the work on the Al-Mg-Ti system [33], the element Mg precipitates from the Al-Mg matrix with increasing temperature and Mg can react with Al and Al_3_Ti, and the reaction formula is as follows:

12Al + 3Mg + 2Al_3_Ti = Al_18_Ti_2_Mg_3_. (2)

The ΔH of this reaction is −50 KJ/mol, and the reaction can occur spontaneously. This is consistent with the experimental phenomenon in this work. The element Mg precipitates from the matrix and changes the L1_2_-Al_3_Ti into Al_18_Ti_2_Mg_3_ completely. Due to the similar properties of Sc and Ti, the Sc atoms replace a part of the Ti atoms in the Al_18_Ti_2_Mg_3_ precipitates, so the final precipitate phase is Al_18_Mg_3_(Sc,Ti)_2_. These Al_18_Mg_3_(Sc,Ti)_2_ precipitates show two different kinds of morphology with different crystal orientations. Additionally, Mn also precipitates in large quantities during the HIP treatment, and Mn does not interact with other elements to form a new phase, still accumulating at grain boundaries.

Compared with the as-fabricated BST2 alloy, the HIPed BST2 alloy has a stronger second-phase strengthening effect. The reinforcement effect can be estimated by the Owen formula [16]:

(3) $$\;\Delta \sigma_{Orowan}=\frac{2G_{m}b}{dp}{\frac{6V_{P}}{\pi }}^{1/3},$$

where *G_m_* is the shear modulus of the matrix, *b* is the Burgers vector, and *d_p_* and *V_P_* are the size of the particles and the volume fraction. The strengthening phase in the HIPed BST2 alloy is mostly Al_18_Mg_3_M_2_(M for Sc,Ti), and the strengthening phase in the as-fabricated BST2 alloy is mostly Al_3_M. The *d_p_* of Al_18_Mg_3_M_2_ is lower than that of Al_3_M, and the *V_p_* of Al_18_Mg_3_M_2_ is higher than that of Al_3_M, according to the TEM results. So Δ*σ_Orowan-Al3M_* should be lower than Δ*σ_Orowan-Al18Mg3M2_*. The hardness of the HIPed BST3 sample is about 15 HV higher than the HIPed BST2 sample, and the average size of the HIPed BST3 sample is about 19% lower than that of the HIPed BST2 sample, but the UTSs of these samples are similar. The hardness and EBSD results come from the cube samples, and the strain–stress curves come from cuboid samples. The cube samples have higher symmetry and are subjected to more uniform force during the HIP treatment. There is a significant deviation between the HIPed cuboid samples. The plasticity of these HIPed samples also improves because of the elimination of pores and unification of organization.

## 4. Conclusions

The Al-Mg-Sc-Ti alloys were fabricated by LPBF in this work, and the role of Ti and Sc elements in the alloys was studied. This work can provide guidance for the design of aluminum alloys for additive manufacturing. Based on the experimental results, the following conclusions can be obtained:

1. Hot cracks in LPBFed Al-Mg alloys can be eliminated by an inoculation treatment with TiH_2_ and ScH_3_. The LPBFed Al-Mg-Sc-Ti alloys exhibit fine exquiaxed grains having a low average grain size. The relative density greatly reduces from 99.8% to 87.8% as the ratio of the Al-Mg raw powders to the composite powders slightly decreases from 100% to 97.7%, and the HIP treatment can heal the pores in the alloys and improve the relative density to nearly 100%.
2. During the LPBF process, Sc and Ti precipitate and react in situ with the matrix. Since Sc and Ti have similar chemical properties, each can replace the other in the in situ reaction, shown by Sc and Ti enriching in the same zones. The reaction produces L1_2_-Al_3_M (M for Sc, Ti) and a small amount of Al_18_M_2_Mg_3_. The L1_2_-Al_3_M serves as a high-quality nucleating agent, and excess Ti and Sc also play a role in limiting grain growth, so that extremely fine equiaxed grain regions form in the as-fabricated alloys.
3. The mechanical properties of the as-fabricated Al-Mg alloys are improved by the co-addition of the elements Ti and Sc. The UTSs of the as-fabricated Al-Mg-0.7Sc-0.7Ti alloy and Al-Mg-0.7Sc-1.0Ti were 275 MPa and 313 MPa, respectively, and the UTSs of the as-fabricated Al-Mg-0.7Sc-1.0Ti and Al-Mg-0.7Sc-1.6Ti were similar. Due to the dehydrogenation reaction, a sample with high Ti addition had low relative density and exhibited poor plasticity.
4. During the HIP treatment, a large amount of Mg precipitates from the matrix, which changes all the in situ Al_3_M into Al_18_M_2_Mg_3_. Alloys undergo grain growth during the HIP treatment, and the element Ti can limit the growth of grains. The hardness and tensile properties of these Al-Mg-Sc-Ti alloys improve significantly after the HIP treatment. The hardness of HIPed Al-Mg-0.7Sc-1.3Ti is 135 HV. The HIPed Al-Mg-0.7Sc-1.0Ti alloy exhibits an UTS of 475 MPa with an El of 8.5%.

## Figures and Tables

**Table 1:** Sample labels of LPBF processed Sc-/Ti-modified Al-Mg alloys.

Sample Labels	TiH_2_ Content	ScH_3_ Content	Al-Mg Content
B	-	-	All
BS	-	0.7	Bal
BST1	0.7	0.7	Bal
BST2	1.0	0.7	Bal
BST3	1.3	0.7	Bal
BST4	1.6	0.7	Bal

**Table 2:** Optimized LPBF processing parameters.

Process Parameter	Value
Hatching spacing	110 μm
Laser power	360 W
Layer thickness	30 μm
Scanning speed	800 mm/s

## Data Availability

Data are contained within the article.
